# Complete transcatheter closure of residual aortopulmonary window in an elderly patient allowing optimization of refractory pulmonary arterial hypertension therapy

**DOI:** 10.1093/ehjcr/ytag773

**Published:** 2026-10-22

**Authors:** Yusuke Akazawa, Takashi Higaki, Osamu Yamaguchi

**Affiliations:** Department of Cardiology, Pulmonology, Hypertension and Nephrology, Ehime University Graduate School of Medicine, Toon, Ehime 791-0295, Japan; Department of Pediatric and Adolescent Therapeutic and Developmental Education, Ehime University Graduate School of Medicine, Toon, Ehime 791-0295, Japan; Department of Pediatrics, Ehime University Graduate School of Medicine, Toon, Ehime 791-0295, Japan; Department of Cardiology, Pulmonology, Hypertension and Nephrology, Ehime University Graduate School of Medicine, Toon, Ehime 791-0295, Japan

**Keywords:** Aortopulmonary window, Pulmonary arterial hypertension, Transcatheter closure, Adult congenital heart disease, Sotatercept

An aortopulmonary window (APW) is a rare congenital heart disease with a left-to-right shunt. When this shunt persists into adulthood and causes pulmonary arterial hypertension (PAH), management becomes challenging. A 73-year-old man had undergone two surgical closures of an APW at ages 17 and 42, but a residual shunt persisted (see Supplementary material online, *Figure S1*). At age 68, worsening heart failure (HF) required hospitalization, during which severe PAH was identified and macitentan was initiated. At our initial evaluation at age 69, WHO functional class III and BNP 175.4 pg/ml suggested an intermediate–high-risk profile based on the available components of the 2022 ESC/ERS four-strata follow-up risk assessment.^1^ Transcatheter closure was then attempted in the setting of severe PAH (mean pulmonary artery pressure 56 mmHg, Qp/Qs 1.6) and right HF (*Figure 1*, Panel *A*), but anatomical constraints allowed deployment of a 7.5-mm Figulla Flex II septal occluder (Occlutech GmbH, Jena, Germany; off-label use), resulting in partial occlusion. At 6 months after partial closure, Qp/Qs had decreased to 1.3 and mean pulmonary artery pressure to 45 mmHg, whereas pulmonary vascular resistance remained elevated (see Supplementary material online, *Table S1*). Given the modest residual shunt, persistent severe PAH, and the patient's preference against further closure, additional intervention was deferred, and PAH-targeted therapy was intensified. At 18 months, Qp/Qs increased to 1.7, accompanied by increased pulmonary blood flow. Subsequently, Qp/Qs declined despite worsening pulmonary hypertension and elevated cardiac filling pressures. Together with recurrent atrial fibrillation and mitral and tricuspid regurgitation, these haemodynamic abnormalities contributed to multifactorial HF requiring continuous intravenous dobutamine and high-dose diuretics (Panel *B*; Supplementary material online, *Videos S1* and *S2*).

**Figure 1:**
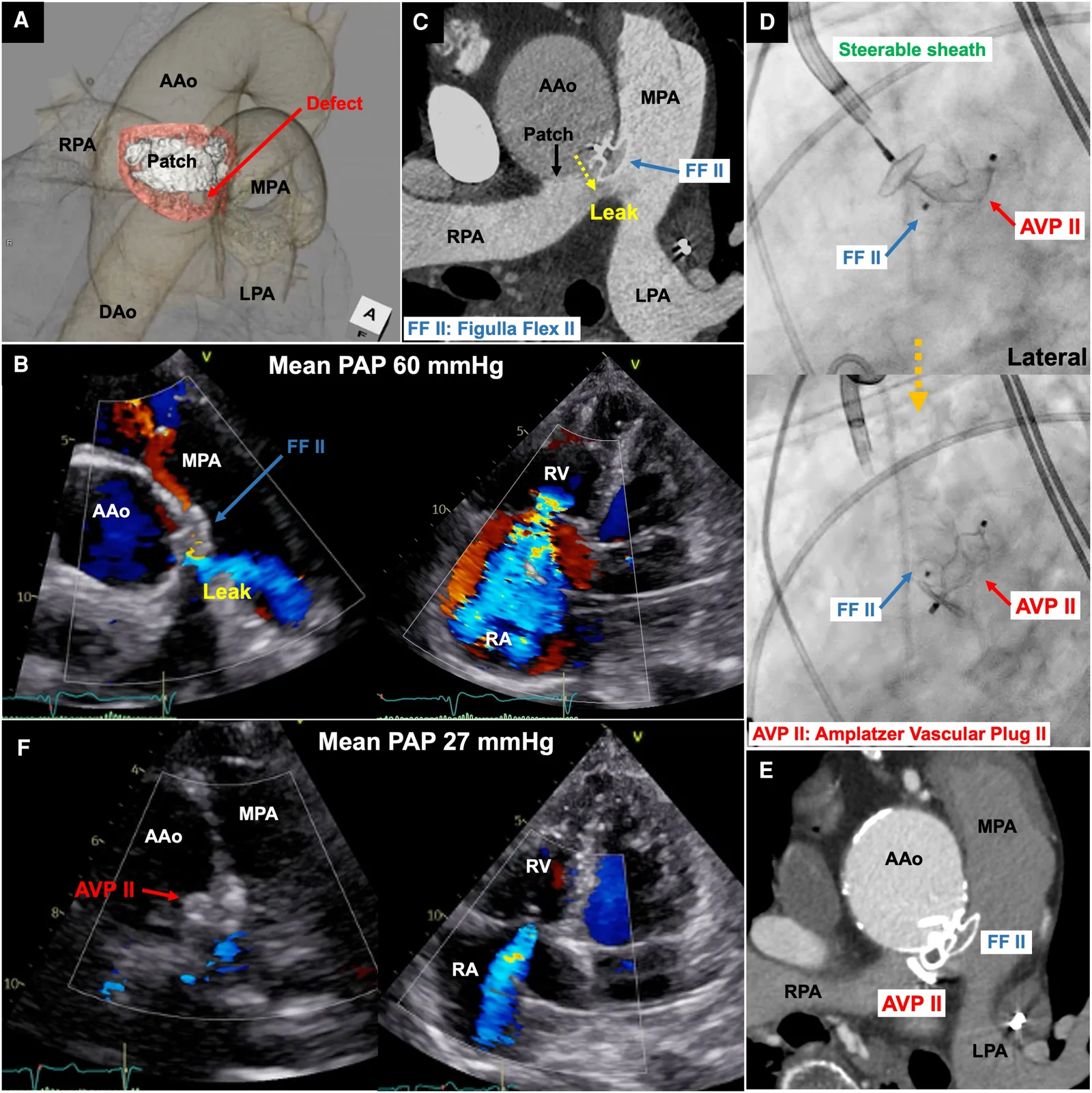
Complete transcatheter closure of a residual aortopulmonary window. (*A*) Three-dimensional computed tomography reconstruction showing the residual defect adjacent to the surgical patch. (*B*) Colour Doppler echocardiography demonstrating residual shunt flow adjacent to the Figulla Flex II device and severe tricuspid regurgitation. (*C*) Computed tomography showing the residual communication between the ascending aorta and right pulmonary artery. (*D*) Fluoroscopic images showing deployment of the Amplatzer Vascular Plug II using a steerable sheath. (*E*) Post-procedural computed tomography showing the two occlusion devices. (*F*) Follow-up echocardiography demonstrating elimination of residual shunt flow and improvement in tricuspid regurgitation. AAo, ascending aorta; DAo, descending aorta; MPA, main pulmonary artery; RPA, right pulmonary artery; LPA, left pulmonary artery; RA, right atrium; RV, right ventricle; FF II, Figulla Flex II; AVP II, Amplatzer Vascular Plug II.

Computed tomography demonstrated APW between the ascending aorta and the right pulmonary artery bifurcation (Panel *C*). Given his age, prior sternotomies and PAH, redo surgery was prohibitive. Instead, a transcatheter strategy using a steerable sheath for coaxial alignment enabled placement of an Amplatzer Vascular Plug II (AGA Medical Corporation, Plymouth, MN, USA; off-label use), achieving complete occlusion (Panels *D* and *E*; Supplementary material online, *Video S3*).

Following complete closure, dobutamine dependence initially persisted, but elimination of the residual shunt facilitated intensification of PAH-targeted therapy, including the addition of sotatercept. As tricuspid regurgitation improved and mean pulmonary artery pressure fell to 27 mmHg, inotropes were discontinued, diuretics were transitioned to oral therapy, and the patient was discharged without surgery (Panel *F*; Supplementary material online, *Videos S4* and *S5*). In selected elderly patients with residual APW, severe PAH, and prohibitive surgical risk, catheter-based closure may facilitate optimization of PAH therapy and improve the management of refractory HF.

## Supplementary Material

ytag773_Supplementary_Data

## Data Availability

All data relevant to this case report are included in the article and its online Supplementary material. Additional patient-level data are not publicly available due to patient confidentiality.
