# Supplementary material for: Complete transcatheter closure of residual aortopulmonary window in an elderly patient allowing optimization of refractory pulmonary arterial hypertension therapy
Source: Eur Heart J Case Rep. 2026 Oct 22;10(10):ytag773. doi: 10.1093/ehjcr/ytag773 (PMC13715630; doi:10.1093/ehjcr/ytag773)
Supplement: Supplementary file 1 [file ytag773_supplementary_data.zip › Figure S1.pdf]

Clinical Course

Infancy	Reportedly diagnosed with valvular heart disease.
17 years	Haemoptysis led to the diagnosis of APW; **first surgical closure** performed.
42 years	Atrial fibrillation (AF) and sick sinus syndrome; permanent pacemaker implantation; residual APW identified and **second surgical closure** performed.
58 years	Follow-up transferred to a local cardiology clinic.
67 years	Pulmonary vein isolation for AF.
68 years	Worsening heart failure requiring hospitalization; residual APW and severe PAH identified; macitentan initiated.
69 years	Initial evaluation at our adult congenital heart disease centre. **First transcatheter closure** using Figulla Flex II; residual shunt remained.
73 years	Dobutamine-dependent heart failure; **second transcatheter closure** using AVP II; PAH therapy intensification including sotatercept, followed by dobutamine withdrawal.

APW, aortopulmonary window; AF, atrial fibrillation; PAH, pulmonary arterial hypertension; AVP II, Amplatzer Vascular Plug II.
