# Supplementary material for: Complete transcatheter closure of residual aortopulmonary window in an elderly patient allowing optimization of refractory pulmonary arterial hypertension therapy
Source: Eur Heart J Case Rep. 2026 Oct 22;10(10):ytag773. doi: 10.1093/ehjcr/ytag773 (PMC13715630; doi:10.1093/ehjcr/ytag773)
Supplement: Supplementary file 1 [file ytag773_supplementary_data.zip › Supplemental Table S1.pdf]

Supplementary Table S1. Serial haemodynamic measurements

Parameter	Before 1st closure	6 months after 1st	18 months after 1st	4 years after 1st	Before 2nd closure	During 2nd closure (general anaesthesia)	2 months after 2nd
**A. Serial right heart catheterization and intra-procedural measurements**							
**Cardiac rhythm**	AF	Sinus rhythm	Sinus rhythm	AF	AF	AF	Sinus rhythm
**PAH-targeted therapy**	Macitentan 10 mg/day	Macitentan 10 mg/day	Macitentan 10 mg/day<br>Selexipag 0.8 mg/day	Macitentan 10 mg/day<br>Selexipag 2.0 mg/day	Macitentan 10 mg/day<br>Selexipag 2.0 mg/day	Macitentan 10 mg/day<br>Selexipag 2.0 mg/day	Macitentan 10 mg/day<br>Selexipag 2.8 mg/day<br>Riociguat 1.5 mg/day<br>Sotatercept 0.7 mg/kg every 3 weeks
**Dobutamine (μg/kg/min)**	—	—	—	—	3	3	—
**Mean RAP (mmHg)**	10	7	7	15	20	11	4
**Mean main PAP (mmHg)**	56	45	52	55	60	42 → 36 (After closure)	27
**PAWP (mmHg)**	18	15	10	24	26	20	12
**Qp index (L/min/m²)**	4.1	3.2	3.8	2.5	3.3	—	3.4
**Qs index (= systemic CI) (L/min/m²)**	2.6	2.4	2.3	2.1	3.9	—	3.4
**Qp/Qs**	1.6	1.3	1.7	1.2	0.9	—	1.0
**Systemic CO (L/min)**	3.6	3.4	3.2	2.9	5.5	—	4.8
**PVR (WU)**	6.6	6.8	7.9	8.9	7.4	—	3.1
**PVRI (WU·m²)**	9.3	9.5	11.1	12.4	10.3	—	4.4
**B. Acute oxygen and inhaled nitric oxide challenge (O_2 15 L/min + NO 20 ppm)**							
**Mean main PAP (mmHg)**	47	40	36	49	—	—	—
**PAWP (mmHg)**	21	16	12	25	—	—	—
**Qp/Qs**	2.4	2.1	1.6	1.5	—	—	—
**PVR (WU)**	3.0	2.6	3.6	5.2	—	—	—

Abbreviations

AF, atrial fibrillation; CI, cardiac index; PAH, pulmonary arterial hypertension; PAP, pulmonary artery pressure; PAWP, pulmonary artery wedge pressure; PVR, pulmonary vascular resistance; PVRI, indexed pulmonary vascular resistance; Qp, pulmonary blood flow; Qs, systemic blood flow; RAP, right atrial pressure; WU, Wood units.
